# Visualization of cholesterol deposits in lysosomes of Niemann-Pick type C fibroblasts using recombinant perfringolysin O

**DOI:** 10.1186/1750-1172-9-64

**Published:** 2014-04-28

**Authors:** Katarzyna Kwiatkowska, Ewelina Marszałek–Sadowska, Gabriela Traczyk, Piotr Koprowski, Małgorzata Musielak, Agnieszka Ługowska, Magdalena Kulma, Anna Grzelczyk, Andrzej Sobota

**Affiliations:** 1 Department of Cell Biology, Nencki Institute of Experimental Biology, 3 Pasteur St., 02-093 Warsaw, Poland; 2 Department of Genetics, Institute of Psychiatry and Neurology, 9 Sobieskiego St., 02-957 Warsaw, Poland; 3 Institute of Biochemistry and Biophysics, 5a Pawinskiego St., 02-106 Warsaw, Poland

**Keywords:** Niemann-Pick C disease, Lipid storage diseases, Cholesterol-binding proteins, Filipin, Perfringolysin O

## Abstract

**Background:**

Niemann-Pick disease type C (NPC) is caused by defects in cholesterol efflux from lysosomes due to mutations of genes coding for NPC1 and NPC2 proteins. As a result, massive accumulation of unesterified cholesterol in late endosomes/lysosomes is observed. At the level of the organism these cholesterol metabolism disorders are manifested by progressive neurodegeneration and hepatosplenomegaly. Until now filipin staining of cholesterol deposits in cells has been widely used for NPC diagnostics. In this report we present an alternative method for cholesterol visualization and estimation using a cholesterol-binding bacterial toxin, perfringolysin O.

**Methods:**

To detect cholesterol deposits, a recombinant probe, perfringolysin O fused with glutathione S-transferase (GST-PFO) was prepared. GST-PFO followed by labeled antibodies or streptavidin was applied for immunofluorescence and immunoelectron microscopy to analyze cholesterol distribution in cells derived from NPC patients. The identity of GST-PFO–positive structures was revealed by a quantitative analysis of their colocalization with several organelle markers. Cellular ELISA using GST-PFO was developed to estimate the level of unesterified cholesterol in NPC cells.

**Results:**

GST-PFO recognized cholesterol with high sensitivity and selectivity, as demonstrated by a protein/lipid overlay assay and surface plasmon resonance analysis. When applied to stain NPC cells, GST-PFO decorated abundant deposits of cholesterol in intracellular vesicles that colocalized with filipin-positive structures. These cholesterol deposits were resistant to 0.05%-0.2% Triton X-100 used for cells permeabilization in the staining procedure. GST-PFO-stained organelles were identified as late endosomes/lysosomes based on their colocalization with LAMP-1 and lysobisphosphatidic acid. On the other hand, GST-PFO did not colocalize with markers of the Golgi apparatus, endoplasmic reticulum, peroxisomes or with actin filaments. Only negligible GST-PFO staining was seen in fibroblasts of healthy individuals. When applied to cellular ELISA, GST-PFO followed by anti-GST-peroxidase allowed a semiquantitative analysis of cholesterol level in cells of NPC patients. Binding of GST-PFO to NPC cells was nearly abolished after extraction of cholesterol with methyl-β-cyclodextrin.

**Conclusions:**

Our data indicate that a recombinant protein GST-PFO can be used to detect cholesterol accumulated in NPC cells by immunofluorescence and cellular ELISA. GST-PFO can be a convenient and reliable probe for revealing cholesterol deposits in cells and can be useful in diagnostics of NPC disease.

## Background

Niemann-Pick disease type C (NPC) is a lysosomal storage disorder caused by accumulation of unesterified cholesterol in cells of the brain, liver, etc., that occurs with an estimated frequency of 1 in 120 000 individuals [1-4]. This value can be underestimated, since atypical phenotypes may not be properly diagnosed [4]. Cholesterol is internalized by cells from serum mainly as a constituent of low density lipoprotein (LDL) by clathrin-mediated endocytosis. The LDL is directed to lysosomes where it is hydrolyzed and free unesterified cholesterol is released [5]. The cholesterol is transported from lysosomes to the plasma membrane and the endoplasmic reticulum where it undergoes esterification. Simultaneously, *de novo* synthesis of cholesterol and LDL uptake are down-regulated [2,6,7].

The NPC disease is caused by mutations of *NPC1* or *NPC2* genes coding for lysosomal proteins – NPC1 and NPC2. About 95% of NPC cases are linked to mutations in the *NPC1* gene [8,9]. NPC1 is a transmembrane lysosomal protein while NPC2 is localized in the lumen of lysosomes [10,11]. The NPC1 and NPC2 proteins are engaged in transporting free cholesterol and some accompanying glycolipids from lysosomes to other cellular compartments [6,12,13]. In addition to cholesterol accumulation in lysosomes its synthesis and metabolism are also affected leading to disturbances in the synthesis of steroid hormones and in the assembly of cellular membranes. Predominant symptoms of NPC disease are progressive neurodegeneration and hepatosplenomegaly. The severity of symptoms of NPC disease varies, but typically the disease leads to death in the second decade of life [3,14]. The neuropathological lesions in NPC patients can be reduced by application of an inhibitor of glucosylceramide synthase, the main enzyme involved in glycosphingolipid synthesis [15].

Presently, detection of NPC disease requires skin biopsy, cultivation of fibroblasts and their staining with filipin, a fluorescent polyene antibiotic which binds free cholesterol [3,16]. However, this approach requires UV excitation and filipin fluorescence is prone to photobleaching, which constrains its application in NPC diagnostics [17,18]. Other methods of NPC diagnosis are also considered [19]. Recently, a new approach for detection of NPC disease based on LC-MS/MS analysis of oxidized forms of cholesterol in the serum has been proposed [20], but a wider application of this sensitive and specific method is limited by the availability of the sophisticated equipment.

Alternative visualization of cholesterol deposits in NPC cells could in principle be also based on the application of protein toxins of microbial origin which specifically recognize free cholesterol and can be used as probes for cell staining without the drawbacks of filipin. About twenty toxins produced by Gram-positive bacteria belong to the family of cholesterol-binding cytolysins [21,22]. Among such bacterial toxins special attention has been paid to perfringolysin O (PFO) produced by *Clostridium perfringens*[23,24]. PFO oligomerizes upon binding to membrane cholesterol and leads to pore formation provided the cholesterol content exceeds 30 mol% [25,26]. A biotinylated proteolytic derivative of PFO, named BCθ, of molecular mass of 57 kDa, has been used to stain NPC cells. In those studies, cells were fixed with 4% paraformaldehyde and exposed to BCθ without cellular membrane permeabilization, yielding intracellular staining [27,28]. Taking into account the relatively high molecular mass of BCθ, the mechanism of its entry into cells and the nature of “the cholesterol-rich domains” detected by BCθ [27] remain unknown.

In this study we prepared PFO fused with glutathione S-transferase (GST) and used the recombinant protein to detect cholesterol-rich organelles in Triton X-100-permeabilized NPC and healthy-donor cells. The GST-PFO-positive vesicles, found only in NPC cells, also stained with filipin, antibodies against lysosomal-associated membrane protein 1 (LAMP-1) and lysobisphosphatidic acid (LBPA), indicating that the procedure detected accumulation of cholesterol in late endosomes/lysosomes in NPC cells.

## M**ethods**

### Fibroblast cultures

The studies were performed on fibroblasts derived from NPC patients and from healthy donors. The NPC patients had been diagnosed on the basis of clinical, cytochemical (Department of Genetics, Institute of Psychiatry and Neurology, Warsaw, Poland) and/or molecular parameters (NZOZ Genomed, Warsaw, Poland). To obtain fibroblast cultures, skin biopsies were dissected and cells were grown for up to 7 passages in DMEM medium containing 10% FBS, 50 U/ml penicillin, 50 μg/ml streptomycin, 0.2 μg/ml amphotericin B at 37°C, 5% CO_2_. Prior to experiments cells were transferred to DMEM medium with delipidated 10% FBS and cultured for 72 h [29]. The delipidated FBS was prepared according to Cham and Knowles [30] using organic solvents (di-isopropyl ether and n-butanol, 40:60, v:v) and filtered throughout a 0.22 μm sterile filter. The procedure removed 96% of the original cholesterol content in FBS, as determined by the Cholesterol DST kit (Alpha Diagnostics). This study was conducted in accordance with the Helsinki Declaration. Ethical approval of the study was requested at the local Bioethical Committee of the Institute of Psychiatry and Neurology in Warsaw, Poland. According to the information obtained from the Committee, the usage of biological material taken from patients, which was excessive after diagnostics procedures, does not demand a special approval on the condition that patients signed an informed consent for such action. Patients gave their signed informed consent for the use of their cells in the scientific experiments after anonimization of the material samples.

For cholesterol depletion, cells cultured in medium containing delipidated 10% FBS were fixed, permeabilized as described further, and exposed for 1 h to 3 mM methyl-β-cyclodextrin (Sigma) in PBS at 37°C. To trigger uptake of LDL, cells were cultured in the presence of delipidated 10% FBS (72 h) and subsequently incubated with 10 μg/ml DiI-LDL (Biomed. Technology) in DMEM/delipidated 10% FBS for 5 h at 37°C.

### Preparation of recombinant PFO

A synthetic gene for perfringolysin O of *Clostridium perfringens* was prepared by GenScript (USA) basing on cDNA sequence No. CP000246.1 at NCBI. The sequence was optimized for expression in *E. coli*. The synthesized gene was devoid of a leader sequence coding for 28 N-terminal amino acids to ensure intracellular accumulation of the expressed protein. The product was cloned by the vendor into pUC57 plasmid. To obtain PFO with a GST tag at the N-terminus, the construct was cloned into pGEX4T vector using BamHI and EcoRI sites. To allow potential removal of the GST tag, the construct was modified by introducing the sequence 5′ GAA AAC CTG TAT TTT CAG GGC 3′ encoding the ENLYFQG motif recognized by Tobacco Etch Virus (TEV) protease [31].

The recombinant vector was transformed into BL-21 (DE3) strain of *E. coli*. Bacteria were grown at 37°C in LB medium containing 100 μg/ml ampicillin to OD = 0.6, when 0.5 mM IPTG was added. The culture was continued at 18°C for 20 h, bacteria were harvested, washed in PBS and lysed in the presence of 0.35 mg/ml lysozyme (10 min, 4°C). The obtained suspension was supplemented with 1% Triton X-100 and sonicated on ice for 15 min at 0.3 cycle, amplitude 33%, using an UP200S Hielscher sonifier (Germany). The lysate was clarified by centrifugation at 20 000 × g for 40 min at 4°C and loaded onto a Glutathione-Sepharose 4B column (bioWORLD). GST-PFO was eluted from the column with 10 mM glutathione, 10 mM DTT, 50 mM Tris, pH 8.0. The GST protein was prepared as described earlier [32]. The presence and purity of the recombinant proteins in column fractions were examined by 10% SDS-PAGE. Fractions containing highest amounts of GST-PFO or GST were pooled and filtered over Amicon Ultra-15 centrifugal filter units to remove glutathione and concentrate the protein. Samples containing about 0.45 mg/ml GST-PFO or 0.6 mg/ml GST in 5 mM DTT, 50 mM Tris, pH 8.0 were supplemented with 20% sucrose and frozen in liquid nitrogen.

### Protein-lipid overlay assay

The analysis was performed essentially as described earlier [33] with modifications. The following lipids were used: semisynthetic bovine brain sphingomyelin (SM), C16-ceramide, cholesterol, dioleoylphosphatidylcholine (DOPC), dipalmitoylphosphatidylcholine (DPPC) and dipalmitoylphosphatidylethanolamine (DPPE) (all from Sigma). The lipids were dissolved in chloroform:methanol:H_2_O (1:1:0.3) and 1 μl of the solution containing 25–500 pmoles of lipid was spotted onto a 0.45-μm nitrocellulose membrane. The membrane was pressed with a hot block at 60°C for 5 s according to Taki and Ishikawa [34], blocked with 1% gelatin and 1% polyvinylpyrrolidone and incubated for 45 min at 20°C with 1 μg/ml GST-PFO in PBS buffer containing 0.03% Tween-20. After washing, the membrane was exposed for 45 min to chicken anti-GST IgY-peroxidase (Rockland). Immunoreactive spots were visualized with the SuperSignal West Pico chemiluminescence substrate (Pierce).

### Carboxyfluorescein release from liposomes

Liposomes composed of (i) cholesterol:SM:DOPC (mol% 40:30:30), (ii) cholesterol:DPPE:DOPC (mol% 40:30:30), (iii) SM:DPPC:DOPC (mol% 40:30:30) or (iv) DPPE:DPPC:DOPC (mol% 40:30:30) loaded with 10 mM 6-carboxyfluorescein were prepared as described earlier [32] with modifications. Lipids were mixed, dried under nitrogen, resuspended in PBS containing 10 mM 6-carboxyfluorescein and sonicated in nitrogen atmosphere (15 min, 4°C, 0.3 cycle with amplitude 33% in the UP200S Hielscher sonifier). Pelleted liposomes (2000 × g, 10 min) were resuspended in PBS at a total concentration of lipids of 1 mM. Release of 6-carboxyfluorescein from the liposomes was induced by 15 μg/ml GST-PFO and measured at Em/Ex = 490/520 nm on a Spex spectrofluorimeter (Jobin-Yvone). The maximal efflux of 6-carboxyfluorescein was determined in the presence of 0.2% Triton X-100.

### Surface plasmon resonance (SPR)

The analysis was performed on large unilamellar vesicles, 100 nm in diameter, containing cholesterol:DOPC, DPPE:DPPC or SM:DPPC (mol% 50:50) and prepared as described by Kulma et al. [32]. The liposomes were deposited on the L1 chip of a BIACore X apparatus (BIACore, GE Healthcare) at a flow rate of 1 μl/min for 10 min. Binding experiments were performed at 10 μg/ml GST-PFO or 10–60 μg/ml GST in 150 mM NaCl, 30 mM Tris, pH 8.0, at a flow rate of 30 μl/min. After 10 min (binding phase) the samples were washed for another 10 min (dissociation phase).

### Determination of total cellular cholesterol

Cells, 3×10^6^/sample, were scraped from dishes after 72 h of culturing in DMEM/delipidated 10% FBS, washed twice with PBS, suspended in 200 μl of hexane:isopropanol (3:2, v:v), sonicated and incubated for 15 min at 20°C with shaking. Extracted lipids were dried under N_2_ and resuspended in 50 μl isopropanol (45 min, 20°C). Aliquots of 30 μl were mixed with 300 μl of Cholesterol DST reagent and processed according to the manufacturer’s instructions (Alpha Diagnostics). Protein content in “cell skeletons” remaining after lipid extraction was estimated by Bradford Ultra reagent (Expedeon Ltd) and measured at 595 nm.

### Determination of chitotriosidase activity

The enzyme activity was measured in samples of blood serum using 4-methylumbelliferyl-β-D-*N,N,N’*-triacetylchitotriose as a substrate (Sigma) according to [35].

### Cellular ELISA

Cells were seeded in DMEM/10% FBS on 96-well plates at 5×10^3^ cells/well, unless indicated otherwise. After 8 h, the medium was replaced with DMEM/delipidated 10% FBS and the cultures were grown for 72 h. Then, the cells were washed, fixed in 3% paraformaldehyde in PHEM buffer (60 mM PIPES, 25 mM HEPES, 10 mM EGTA, 4 mM MgCl_2_, pH 6.9), washed in PBS buffer, exposed to 50 mM NH_4_Cl/PBS (5 min, 20°C), washed again and permeabilized with 0.05% Triton X-100/PBS for 10 min at 20°C. In a series of experiments cells were permeabilized with 0.03-0.2% Triton X-100 (10 min, 20°C) or 0.05% digitonin (10 min, 20°C). The detergent was washed out with PBS and the cells were incubated for 45 min at 20°C in 3% fish gelatin/PBS followed by 5 μg/ml GST-PFO in PBS buffer containing 1% fish gelatin (Sigma). When indicated, prior to labeling with GST-PFO cells were incubated with 3 mM methyl-β-cyclodextrin for 60 min at 37°C. After washing 5 times in 1% fish gelatin/PBS, the cells were incubated with 2 μg/ml chicken anti-GST IgY-peroxidase in 1% fish gelatin/PBS (45 min, 20°C), washed 3 times in 1% fish gelatin/PBS and 3 times in PBS alone. The enzymatic activity of bound peroxidase was examined in the presence of 0.05 mg/ml 3,3′,5,5′-tetramethylbenzidine and 0.045% H_2_O_2_. After 30 min, the reaction was stopped with 1 M H_2_SO_4_ and the absorbance was measured at 450 nm using a Sunrise Plate Reader (Tecan Group). The results were normalized against protein content in samples estimated by Bradford Ultra at 595 nm. Samples were run in parallel with and without treatment with GST-PFO. The values of 450/595 absorbance obtained in samples not-treated with GST-PFO were subtracted from the corresponding values of samples incubated with GST-PFO.

### Immunofluorescence studies

Cells (1×10^4^/sample) were seeded on coverslips (15×15 mm) in DMEM containing 10% FBS and after 18 h the medium was replaced with DMEM supplemented with delipidated 10% FBS and cultured for 72 h. Cells were washed with PBS, fixed with 3% paraformaldehyde in PHEM buffer (30 min, 20°C) and processed essentially as described [36]. Briefly, cells were washed, incubated with 50 mM NH_4_Cl/PBS for 5 min at 20°C and permeabilized with 0.05% Triton X-100/PBS for 10 min at 4°C. Alternatively, in a series of experiments cells were permeabilized with 0.05% digitonin/PBS for 10 min at 20°C. After washing in PBS, cells were incubated twice with 3% fish gelatin in PBS (30 min each incubation). Next, cells were treated with 5 μg/ml GST-PFO for 45 min at 20°C and washed five times in 0.2% fish gelatin/PBS. To detect the probe, the cells were exposed to goat IgG anti-GST conjugated with biotin (Rockland) prepared in 0.2% fish gelatin/PBS (45 min, 20°C). In studies on colocalization of PFO-stained structures, the anti-GST antibody was accompanied by: (i) rabbit anti-LAMP-1 IgG (Santa Cruz Biotechnology), (ii) rabbit anti-golgin-84 IgG (Santa Cruz Biotechnology), (iii) rabbit anti-protein disulfide isomerase (PDI; Cell Signaling), (iv) mouse anti-peroxisomal membrane protein 70 (PMP70) (Sigma), (v) mouse anti-LBPA IgG (clone 6C4; Echelon), or (vi) phalloidin-FITC (Sigma). Unbound antibodies and phalloidin were washed out five times with 0.2% fish gelatin/PBS and the samples were incubated for 45 min at 20°C either with streptavidin-TRITC (Sigma) and goat anti-rabbit IgG F(ab)_2_-FITC (Jackson ImmunoResearch) or goat anti-mouse IgG-FITC (ICN). In some experiments cells were exposed to 25 μg/ml filipin III (Sigma) for 45 min at 20°C in darkness and GST-PFO was detected using goat IgG anti-GST followed by donkey anti-goat-Texas Red (Jackson Immunoresearch). When living cells were exposed to 10 μg/ml DiI-LDL, they were washed after 5 h at 37°C, fixed with 3% paraformaldehyde, permeabilized with 0.05% Triton X-100, incubated with 5 μg/ml GST-PFO followed by the anti-GST-biotin antibody, as described above and exposed to streptavidin-FITC (45 min, 20°C; Sigma). After extensive washing in 0.2% fish gelatin/PBS, samples were mounted in mowiol/DABCO and examined either under a Nikon microscope equipped with a DXM1200C digital camera or under a Leica confocal microscope (TCS SP8 SMD). TRITC and FITC, TRITC and filipin, DiI-LDL and FITC were excited in the mode of sequential excitation to exclude cross-over of their fluorescence. Stacks of 8–10 confocal planes were acquired for each analyzed cell. The setting of photomultipliers were adjusted to obtain comparable ranges of pixel intensity in each channel, scan resolution was 1024×1024. Colocalization analysis was performed on single-plane confocal images using Leica Application Suite AF software which calculated the Perason’s correlation coefficient and the overlap coefficient [32]. For both signals the intensity threshold value was set at 55% and 20% background subtraction was applied. At least 20 cells from two independent experiments were analyzed for each variant.

### Electron microscopy

For the studies, NPC and control healthy fibroblasts were grown in 10-cm Petri dishes to confluence and after washing with PBS, were fixed with 3% formaldehyde/0.5% glutaraldehyde in 100 mM sodium phosphate buffer (pH 7.2) for 30 min at 20°C. Cells were washed twice with the phosphate buffer, treated with 50 mM NH_4_Cl in the buffer (10 min, 20°C), washed and gently scraped off the dishes. Pelleted cells (2000 × g, 2.5 min) were dehydrated in an ethanol series (20°C) followed by incubations in mixtures of LR White resin/ethanol at ratios 1:1, 2:1 and 3:1, each for 1 h. Finally, the samples were infiltrated with 100% LR White overnight, after which the resin was exchanged twice (1 h, 20°C). The samples were polymerized at 56°C for 48 h. Ultrathin sections were placed on formvar-coated nickel grids and blocked with 3% fish gelatin in PBS and next in a mixture of 3% BSA and 1% polyvinylpyrrolidone in PBS (50 min each incubation). Subsequently, the samples were incubated overnight with 10 μg/ml GST-PFO and rabbit anti-LAMP-1 antibody in 0.2% fish gelatin/PBS in a humid atmosphere. After washing five times with 0.2% fish gelatin/0.05% Tween-20/PBS, the samples were incubated with goat anti-GST IgG-biotin (3.5 h) and after washing, exposed to goat anti-biotin IgG conjugated with 6 nm gold particles and donkey anti-rabbit IgG-10 nm gold (both Aurion) prepared in 0.2% fish gelatin/0.05% Tween-20/PBS (3 h). After extensive washing: six times with 0.2% fish gelatin/0.05% Tween-20/PBS, thrice with PBS and twice with distilled H_2_O, the samples were counterstained with 2.5% uranyl acetate in 50% ethanol for 15 min in dark, washed with 50% ethanol and distilled H_2_O, and stained with lead citrate for 2 min. Finally, the samples were washed with distilled H_2_O, dried and examined under a JEM 1400 (Jeol) electron microscope.

## Results

### Expression of GST-PFO

In order to obtain a selective cholesterol-binding probe, a synthetic gene encoding perfringolysin O was cloned in-frame with the GST-encoding sequence in the pGEX4T vector. To preclude secretion of the fusion protein, the PFO sequence was trimmed at the 5′ end by removing the first 28 amino acids comprising a leader peptide. In addition, a heptapeptide motif recognized by TEV protease was introduced between the GST and 28ΔPFO sequence allowing potential cleavage of the GST tag. The GST-PFO fusion protein was expressed in *E. coli* and purified by one-step affinity chromatography on Glutathione-Sepharose; the protein migrated as a 78-kDa band on SDS-PAGE and was ca. 98% pure (not shown).

**Figure 1:**
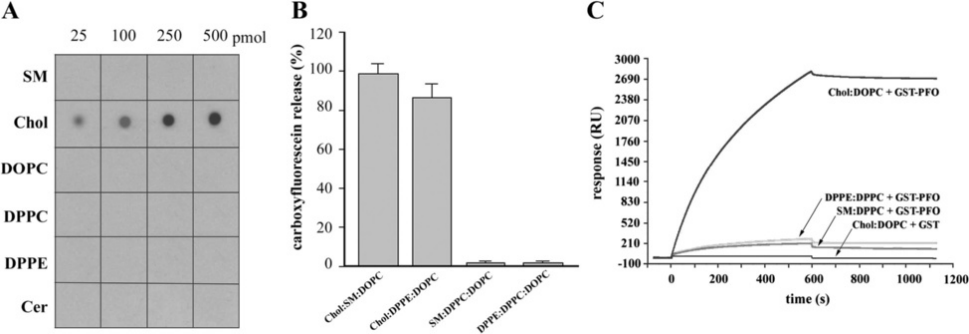
**Selective recognition of cholesterol by GST-PFO. (A)** Protein-lipid overlay assay. Indicated amounts of lipids were spotted on nitrocellulose membrane and incubated with 1 μg/ml GST-PFO. Immunoreactive spots were revealed by chemiluminescence. **(B)** 6-Carboxyfluorescein release from liposomes of indicated composition induced by 15 μg/ml GST-PFO. The data are expressed as percentage of the total amount of 6-carboxyfluorescein released from liposomes by 0.2% Triton X-100. **(C)** SPR sensograms of binding of 10 μg/ml GST-PFO or 20 μg/ml GST to liposomes of indicated lipid composition immobilized on the surface of sensor chip L1. Binding of GST-PFO to liposomes was carried out for 600 s after which the samples were washed for another 600 s. In **(A)** and **(C)** data represent one of three independent experiments; in **(B)** mean ± SEM from three experiments is shown. Chol – cholesterol; Cer – ceramide; SM – sphingomyelin.

In this study the uncleaved GST-PFO protein was used as the GST tag was useful for detection of PFO in cells. To assess whether the recombinant GST-PFO protein selectively recognized and bound cholesterol, a protein-lipid overlay assay was performed. The probe at 1 μg/ml detected cholesterol in a dose-dependent manner starting from 25 pmoles of the lipid and with high specificity, as it did not recognize ceramide or phospholipids such as sphingomyelin, DOPC, DPPC and DPPE (Figure 1A). Upon binding to cholesterol-containing membranes, PFO undergoes oligomerization and forms pores responsible for its lytic activity [37]. We found that GST-PFO displayed lytic activity and released 6-carboxyfluorescein trapped in cholesterol-containing liposomes regardless of the composition of the accompanying phospholipids (Figure 1B). The permeabilizing activity of GST-PFO confirmed the protein’s specificity – it did not induce the release of 6-carboxyfluorescein from liposomes devoid of cholesterol (Figure 1B). When incubated with sheep erythrocytes, in which cholesterol constitutes about 30% of the total lipid content [38], GST-PFO induced maximal hemolysis at 200 ng/ml (not shown).

Strong binding of GST-PFO to cholesterol-containing liposomes was also revealed by surface plasmon resonance analysis (Figure 1C). The binding indicated by a resonance value of about 2800 RU was stable during 10 minutes of washing of the samples. There was no binding of GST alone to the liposomes. The binding of GST-PFO to liposomes devoid of cholesterol was negligible (Figure 1C). The data indicate that GST-tagged PFO retained selectivity for cholesterol binding characteristic for native PFO [25,26].

### GST-PFO and filipin stain the same organelles

**Figure 2:**
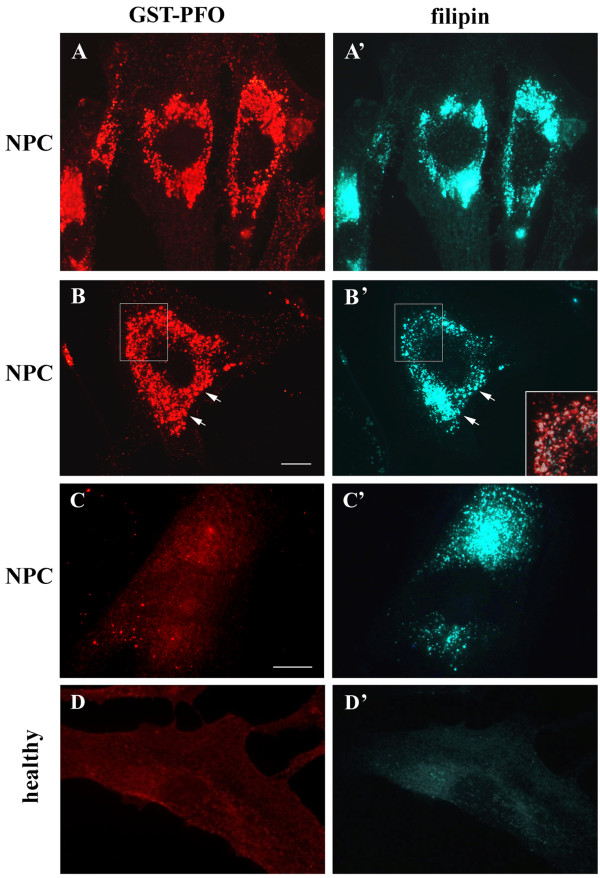
**GST-PFO labels cholesterol deposits in NPC fibroblasts. (A-C)** NPC fibroblasts were cultured in DMEM/10% FBS **(A, A’)** or in DMEM supplemented with delipidated 10% FBS for 72 h **(B-C’)**. Inset in **(B’)** shows an enlargement of a merged image of an area marked in **(B, B’)**. Colocalization of GST-PFO and filipin staining is in white. Colocalization of the labels is also seen in vesicles marked by arrows in **(B, B’)**. **(C, C’)** Omitting GST-PFO during labeling of permeabilized NPC cells yields only traces of non-specific staining with secondary antibodies **(C)**. In these conditions, filipin detects cholesterol accumulated in the cells **(C’)**. **(D-D’)** Fibroblasts from a healthy donor were cultured in DMEM/delipidated 10% FBS. Cells were fixed, permeabilized with 0.05% Triton X-100 and incubated with 5 μg/ml GST-PFO followed by secondary antibodies conjugated with Texas Red (left) or with 25 μg/ml filipin (right) to compare the staining patterns revealed by the two probes. Scale bar, 20 μm.

Filipin staining is a generally accepted tool for detection of cholesterol deposits in NPC cells [3,39-41]. We aimed to characterize PFO as another convenient probe for visualization and analysis of cholesterol in cells. In fibroblasts derived from NPC patients, fixed and permeabilized with 0.05% Triton-X100, GST-PFO strongly stained the abundant perinucler structures (Figure 2A). After prolonged lipid deprivation, the staining was moderately decreased revealing that the GST-PFO-stained deposits were located in vesicles (Figure 2B). The pattern of cell staining by GST-PFO was similar to that shown by filipin (compare Figure 2A and A’, B and B’; typical vesicles with clear colocalization are marked by arrows). However, small vesicles seemed to be decorated more strongly by GST-PFO than by filipin (Figure 2B’, inset). The staining of NPC cells with GST-PFO was specific to the probe since only residual labeling was detected when GST-PFO was omitted. Simultaneously, cholesterol deposits were revealed in these cells by filipin (Figure 2C, C’). Only negligible staining with GST-PFO or filipin was found in fibroblasts from healthy individuals (Figures 2D, D’). Together with the selectivity of GST-PFO for cholesterol demonstrated by physico-chemical approaches presented in Figure 1, the fluorescence microscopy data strongly suggest that GST-PFO detects cholesterol stored in NPC cells.

**Figure 3:**
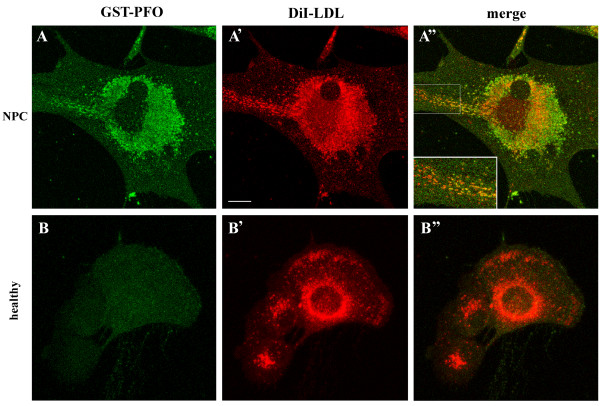
**GST-PFO labels vesicles containing DiI-LDL in NPC cells.** NPC fibroblasts **(A-A”)** and healthy control fibroblasts **(B-B”)** were cultured in DMEM supplemented with delipidated 10% FBS for 72 h, incubated with 10 μg/ml DiI-LDL for 5 h and, after fixation and permeabilization, processed for GST-PFO labeling. **(A, B)** GST-PFO staining, **(A’, B’)** fluorescence of DiI-LDL, **(A”, B”)** merged images. Inset in **(A”**) shows an enlargement of a marked area. Scale bar, 20 μm.

Beside perinuclear cholesterol deposits, GST-PFO probe also detected small vesicles located at the cell periphery which were abundant in NPC cells cultured in a complete medium (Figure 2A). Since these vesicles could correspond to LDL-containing endosomes, NPC cells and fibroblasts from healthy individuals were incubated with 10 μg/ml DiI-LDL for 5 h, and subsequently subjected to GST-PFO co-staining (Figure 3). Confocal microscopy analysis revealed that in NPC cells, DiI-LDL accumulated in various amounts within perinuclear vesicles co-labeled with GST-PFO (Figure 3A-A”). DiI-LDL was also present in numerous vesicles along an “endocytic track” leading from the cell periphery toward the perinuclear region. These vesicles containing DiI-LDL were decorated with GST-PFO with variable intensity: GST-PFO staining was absent in vesicles at the start of the “endocytic track” and increased gradually toward the cell center (Figure 3A”, inset). The presence of free cholesterol in endocytic veiscles can result from disturbances in the lipid distribution in NPC cells or can indicate that in these cells cholesterol deesterification starts already in endosomes. In control fibroblasts, despite intensive LDL endocytosis, no staining of LDL-containing vesicles with GST-PFO was detected (Figure 3B-B”).

### The organelles stained by GST-PFO bear markers of late endosomes and lysosomes

**Figure 4:**
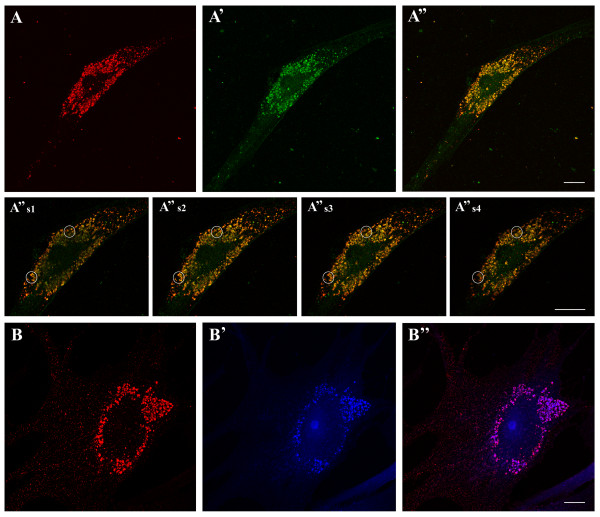
**Colocalization of GST-PFO with LAMP-1 and filipin in NPC fibroblasts.** Cholesterol visualized by GST-PFO (red) is present in lysosomes identified by anti-LAMP-1 (green in **A’-A”**) and filipin (blue in **B’-B”**). **(A”s1-s4)** Merged images of optical sections of the cell shown in **(A-A”)**. The sections range from the basolateral **(A”s1)** to the apical **(A”s4)** part of the cell. **(A”, B”)** Merged images of **(A-A’)** and **(B-B’)**, respectively. Colocalization of GST-PFO and LAMP-1 is seen as yellow color in (A” and A”s1-s4) while pink color reflects colocalization of GST-PFO- and filipin-positive structures in **(B”)**. Scale bars, 20 μm.

**Figure 5:**
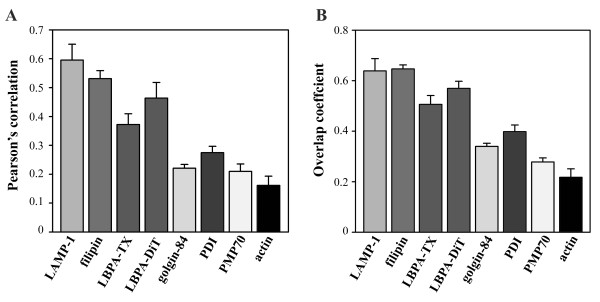
**Quantitation of colocalization of GST-PFO-positive structures and other cellular components in NPC fibroblasts. (A)** Pearson’s correlation coefficient, **(B)** overlap coefficient for co-immunostaining with GST-PFO and filipin or antibodies against LAMP1, LBPA, golgin-84, PDI, PMP70 or phalloidin used as an actin filament marker. TX, Triton X-100, DiT, digitonin. Data shown are mean ± SEM.

To identify the vesicles stained by GST-PFO in NPC fibroblasts, colocalization of the probe with markers of different cellular compartments was analyzed. Of the several organelle-specific probes, the LAMP-1-specific staining of lysosomes overlapped markedly with the GST-PFO signal (Figure 4A-A”). Magnified confocal optical sections revealed that at the basolateral level, LAMP-1 and GST-PFO signals were rather separated but their colocalization increased in the middle part of the cell (Figure 4A” s1-s4). Individual vesicles displayed partial overlapping of LAMP-1 and GST-PFO staining which changed along the vertical axis (Figure 4A” s1-s4, circles). To estimate the degree of colocalization of GST-PFO and LAMP-1-positive vesicles, a quantitative analysis was performed of dual-color confocal images. Two different values were calculated: the Pearson's correlation coefficient and the overlap coefficient [42]. As seen in Figure 5, the Pearson’s correlation coefficient for co-immunostaining of GST-PFO and LAMP was high and reached 0.59 ± 0.05 (in relation to 1 as the maximal value). The overlap coefficient of the two fluorescence patterns was also high (0.64 ± 0.05). We estimated also colocalization of GST-PFO and filipin staining (see Figure 4B-B”). The Pearson’s correlation coefficient and the overlap coefficient for fluorescence generated by these two probes were as high as 0.53 ± 0.03 and 0.65 ± 0.02, respectively (Figure 5).

**Figure 6:**
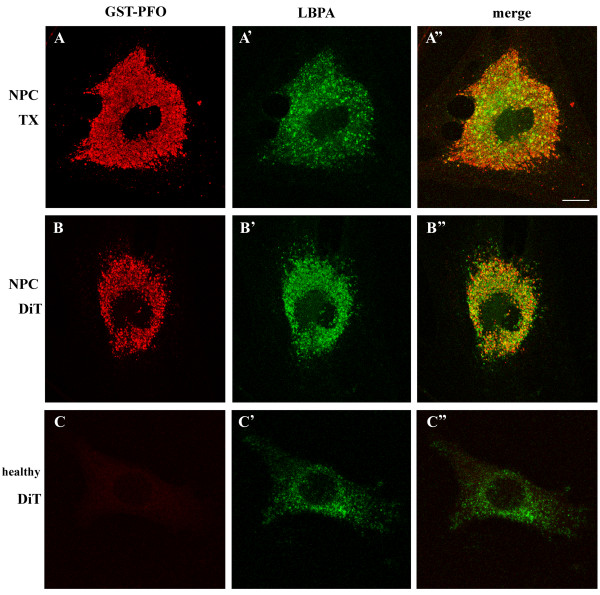
**GST-PFO labeling of NPC cells colocalizes with LBPA.** NPC **(A-B”)** and healthy control fibroblasts **(C-C”)** were fixed, permeabilized with 0.05% Triton X-100 (A-A”) or 0.05% digitonin **(B-C”)** and labeled with GST-PFO and anti-LBPA antibody. **(A, B, C)** GST-PFO labeling, **(A’, B’, C’)** LBPA localization, **(A”, B”, C”)** merged images. TX, Triton X-100, DiT, digitonin. Scale bar, 20 μm.

Previous studies indicated that cholesterol-containing vesicles stained by filipin in NPC cells were rich in LBPA, a marker of late endosomes-multivesicular bodies [43,44]. Our attempts to analyze colocalization of GST-PFO-labeled and LBPA-rich structures were, however, strongly impeded by technical conditions of cell staining. Permeabilization of cells with 0.05% Triton X-100 which was optimal for cholesterol detection (see data below) did not promote visualization of LBPA. In these conditions, only a sub-population of GST-PFO-positive vesicles was labeled with the anti-LPBA antibody (Figure 6A-A”). The Pearson’s correlation coefficient and the overlap coefficient for the two lipids reached 0.36 ± 0.04 and 0.52 ± 0.04, respectively (Figure 5). In accordance, permeabilization of control fibroblast with Triton X-100 did not permit detection of LBPA in these cells (not shown). Application of 0.05% digitonin for cell permeabilization led to detection of numerous LBPA-bearing endosomes in the control cells (Figure 6C-C”), markedly improved detection of LBPA in NPC cells (Figure 6B-B”), and increased the extent of colocalization of LBPA and GST-PFO staining (Figure 5). It should be noted that treatment of cells with digitonin removed substantial amounts of cholesterol from NPC cells (see data below) and could affect colocalization studies. Taken together, the data suggest that vesicles containing cholesterol deposits detected by GST-PFO also contained LAMP-1 and LBPA, the markers of late endosomes and lysosomes.

**Figure 7:**
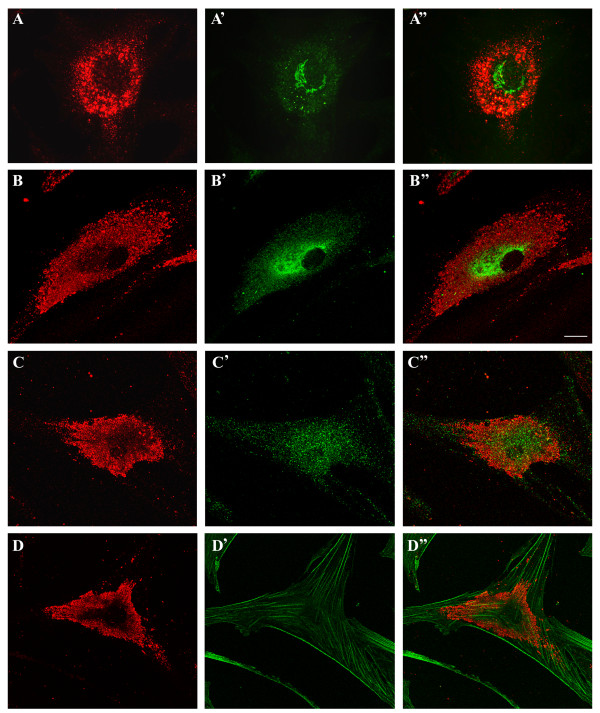
**Negligible co-immunostaining of GST-PFO-positive structures and other organelles in NPC cells. (A-D)** Distribution of GST-PFO-positive structures, **(A’-D’)** patterns of staining by antibodies directed against golgin-84 **(A’)**, PDI - an endoplasmic reticulum marker **(B’)**, anti-PMP70 – a peroxisomal marker **(C’)** and phalloidin labeling actin filaments **(D’)**. **(A”-D”)** merging of the corresponding pairs of images displays traces of the yellow color indicating different localization of GST-PFO-positive structures and the examined proteins. Scale bar, 20 μm.

In contrast, *cis*-Golgi cisterns decorated with anti-golgin-84 antibody were located only in a close vicinity of the nucleus and could be easily distinguished from the GST-PFO-positive structures (Figure 7A-A”). There were also no similarities between the patterns of staining obtained with GST-PFO and with the anti-PDI antibody which decorated the endoplasmic reticulum (Figure 7B-B”). Moreover, antibodies against PMP70 of peroxisomes and phalloidin-FITC, which labels actin filaments, exhibited unique labeling patterns that were unlike the labeling obtained with GST-PFO (Figure 7C-C” and 7D-D”). The lack of colocalization of GST-PFO-positive vesicles with the indicated organelle markers was reflected by low values of the Pearson’s correlation and the overlap coefficients which were in the range of 0.16-0.27 and 0.21-0.40, respectively (Figure 5).

### Immunoelectron microscopy analysis of NPC cell labeling with GST-PFO

**Figure 8:**
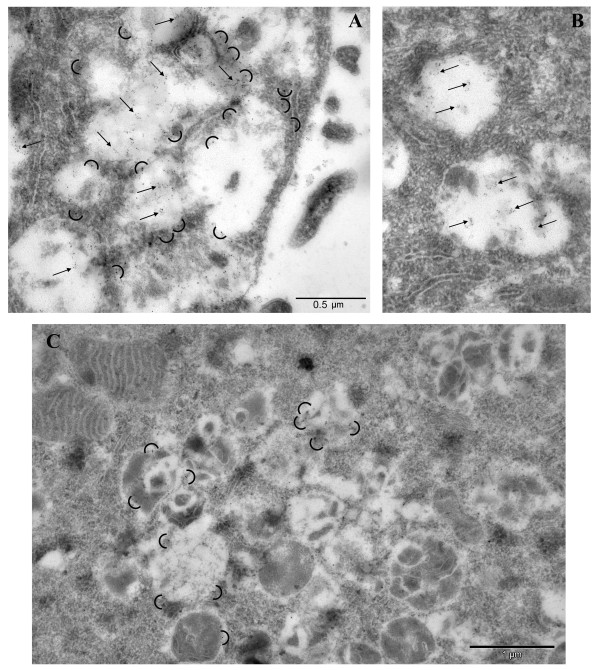
**Immunoelectron microscopy analysis of GST-PFO staining in NPC and healthy fibroblasts.** Ultrathin sections of NPC cells **(A, B)** or control healthy fibroblasts **(C)** were subjected to post-embedding immunogold labeling with GST-PFO followed by anti-GST-biotin and anti-biotin-gold antibodies (6 nm) together with rabbit anti-LAMP-1 and anti-rabbit-gold antibodies (10 nm). GST-PFO decorates material accumulated in vesicles of NPC cells (arrows in **A**, **B**) while LAMP-1 is visualized mainly at the borders of vesicles in NPC and control fibroblasts (semicircles in **A** and **C**).

To perform ultrastructural analysis of GST-PFO labeling, NPC cells were embedded in LR White resin. Ultrastructural sections of the cells were processed for post-embedding labeling with GST-PFO and anti-LAMP-1 antibody. Figure 8A,B shows fragments of NPC cells which contained numerous large vesicles filled with an amorphous material and surrounded by abundant tubular structures. These vesicles and their content as well as their close surroundings were heavily decorated with gold particles attributed to GST-PFO (Figure 8A,B, arrows point to accumulations of 6 nm gold particles). On this basis the electron-opaque content of the vesicles can be ascribed to cholesterol deposits. However, a fraction of cholesterol was probably removed due to harsh conditions used for dehydration and embedding of cells in the resin leaving the interior of some of the lysosomes translucent. The GST-PFO-decorated vesicles were also moderately labeled with anti-LAMP-1 antibody which mainly stained the borders of the structures (Figure 8A, semicircles indicate 10 nm gold particles). This pattern of staining with GST-PFO and anti-LAMP-1 antibody was characteristic to NPC cells. Ultrathin sections of control healthy fibroblasts were devoid of large vesicles and were only scarcely labeled with GST-PFO. LAMP-1-decorating gold particles were found as singlets and small clusters located mainly at the borders of small vesicles (Figure 8C, semicircles).

### Application of GST-PFO for cholesterol determination in NPC cells by cellular ELISA

**Figure 9:**
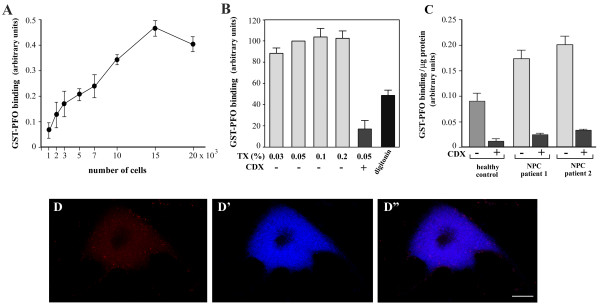
**Determination of cholesterol content in NPC cells by cellular ELISA. (A)** Binding of GST-PFO to cholesterol increases proportionally to the number of cells in the sample within the range of 1-15×10^3^ cells/well. Triton X-100-permeabilized NPC fibroblasts were exposed to 5 μg/ml GST-PFO followed by anti-GST IgY-peroxidase. Absorbance of the product of peroxidase activity is shown. **(B)** Permeabilization of cells with Triton X-100 does not extract cholesterol from deposits. Fixed NPC cells were treated with 0.03%-0.2% Triton-X-100 (TX) or 0.05% digitonin, as indicated, and processed for cellular ELISA using GST-PFO. In a series of experiments, prior to labeling with GST-PFO, cells were incubated with 3 mM methyl-β (CDX) for 60 min at 37°C. In these conditions prominent reduction in the binding of GST-PFO to cells is observed. **(C)** Significant binding of GST-PFO to NPC fibroblasts. NPC cells and healthy controls were processed for cellular ELISA. When indicated, prior to labeling with GST-PFO cells were incubated with 3 mM methyl-β-cyclodextrin. The results are presented as a ratio of absorbance at 450 nm and 595 nm. Data are means ± SEM from four experiments. **(D-D’)** In non-permeabilized cells cholesterol deposits are not stained with GST-PFO **(D)** but are labeled with filipin **(D’)**. **(D”)**: merged images of **D** and **D’**. Scale bar, 20 μm.

The presence of the GST tag in the fusion protein allowed us to estimate the level of free cholesterol in cells by cellular ELISA. The signal generated by GST-PFO/anti-GST IgY-peroxidase increased in a linear manner with the number of cells, from 1×10^3^ to 15×10^3^ cells/well (Figure 9A).

To examine whether Triton X-100 used for permeabilization of cells prior to GST-PFO labeling can extract cholesterol from the deposits, fixed NPC cells were exposed to the detergent in the concentration range from 0.03% to 0.2%. Treatment of cells with 0.05-0.2% Triton X-100 did not significantly affect cholesterol detection by GST-PFO (Figure 9B), indicating that cholesterol deposits in NPC cells are resistant to extraction with these concentrations of detergent. In cells permeabilized with 0.03% Triton X-100 the amounts of cholesterol detected by GST-PFO were slightly lower in comparison to these revealed at 0.05-0.2% Triton X-100 (Figure 9B), suggesting that in these condition the access of the probe to cholesterol deposits could be limited. Finally, GST-PFO failed to label NPC cells that were not permeabilized (Figure 9D). Therefore, permeabilization of cells with Triton X-100 is required for the probe to gain access to the cholesterol deposits in cells. This is in contrast to filipin which stained cholesterol in non-permeabilized cells (Figure 9D’-D”). Moreover, after treatment of cells with 0.05% Triton X-100, cholesterol deposits became sensitive to extraction with 3 mM methyl-β-cyclodextrin which reduced subsequent GST-PFO binding by over 80% (Figure 9B). The data indicate that permeabilization of the plasma membrane and membranes of the intracellular vesicles ensures high efficiency of cholesterol removal from intracellular stores by methyl-β-cyclodextrin. In contrast to Triton X-100, permeabilization of cells with 0.05% digitonin gave unsatisfactory results since it reduced GST-PFO binding by about 50% (Figure 9B).

Using the cellular ELISA based on GST-PFO binding to Triton X-100-permeabilized cells we compared cholesterol levels in fibroblasts from NPC patients and a healthy control. The level of free cholesterol in cells from two patients was fairly similar and was 2–2.4-fold higher than in control fibroblasts (Figure 9C). Consistent with earlier results, exposure of Triton X-100-permeabilized cells to 3 mM methyl-β-cyclodextrin reduced the amount of cholesterol by about 85% in both normal and NPC cells (Figure 9C).

To estimate feasibility of using GSTO-PFO to distinguish between different biochemical phenotypes of NPC cells, the results of PFO-based cellular ELISA were compared with the results of routine filipin staining of cells derived from various NPC patients. Cells obtained from three persons (patients 1–3) unambiguously diagnosed as NPC patients based on clinical symptoms and strong filipin staining of fibroblasts displayed also strong GST-PFO binding in cellular ELISA (Table 1). In addition, the same analysis revealed relatively intense filipin and GST-PFO staining of another fibroblast line (patient 4), despite it was derived from a person whose preliminary NPC diagnosis was not confirmed by genetic testing (Table 1). Of interest, GST-PFO allowed to detect a moderate increase of the free cholesterol level in fibroblasts from a person who was preliminarily classified as a NPC patient based on a weak filipin staining and mild clinical symptoms (patient 5) and the diagnosis was later confirmed by a genetic approach (Table 1). Taken together, the data indicate that GST-PFO can be as useful as filipin in detection of free cholesterol deposits in NPC cells. In comparison, two other potential NPC diagnostic methods, the measurement of total cholesterol content in fibroblasts as well as the chitotriosidase activity in serum of NPC patients yielded much less conclusive results. In general, the total cellular cholesterol content in cells and the chitotriosidase activity in serum were elevated in NPC patients but these parameters did not correlate with the accumulation of free cholesterol in cells or the severity of NPC clinical symptoms (Table 1). This is in agreement with earlier findings that the chitotriosidase activity can change with the age of NPC patients and abnormal activity of the enzyme can be found also in lysosomal storage disorders other than NPC [19,45]. On the other hand, defects in cholesterol esterification, typical for NPC disease, can account for the discrepancies between the total cholesterol level and the accumulation of free cholesterol in NPC cells [39,46].

**Table 1:** Comparisons of different methods used in NPC disease diagnosis

**Patients**	**GST-PFO cellular ELISA (arbitrary units)**	**Filipin staining**	**Total cholesterol content (μg/ml)**	**Chitotriosidase activity (nmol/ml/h)**	**Comments**
1	0.205 ± 0.031	+ + +	115.72 ± 6.06	1515 -1810	Typical symptoms
2	0.192 ± 0.042	+ + +	ND	465 - 783	Typical symptoms
3	0.181 ± 0.024	+ + +	87.21 ± 14.80	212	Typical symptoms
4	0.166 ± 0.036	++	53.86 ± 5.41	ND	Qualified initially as NPC, not confirmed genetically
5	0.130 ± 0.018	+	58.08 ± 11.91	286 - 323	Moderate symptoms - progressing with age, genetically confirmed
Healthy	0.085 ± 0.004	_−_	14.63 ± 1.12	120 - 150	N/A

GST-PFO binding, filipin labeling, and total cholesterol content was estimated in cultured skin fibroblasts, while the chitotriosidase activity in serum of NPC patients and a healthy control. Results of two or three assays run in triplicates are shown as mean ± SEM when indicated.

## Discussion

Mutations in the *NPC1* and *NCP2* genes disturb the trafficking of cholesterol in cells. These mutations are linked to a wide clinical spectrum of NPC disease and their diversity seems to contribute to the heterogeneity of NPC symptoms that range from severe to mild neurological defects, organomegaly and psychiatric symptoms [3,14]. At the cellular level the abnormalities in cholesterol trafficking are manifested by accumulation of free unesterified cholesterol and disturbance in the metabolism of some other lipids, particularly sphingolipids [6,12,47]. At present, cholesterol deposits in NPC cells are detected by staining of cells derived from skin biopsies with the polyene antibiotic filipin [3,16,39].

In this report we demonstrate that cholesterol deposits in fibroblasts from NPC patients can be conveniently visualized using a recombinant bacterial toxin, perfringolysin O fused with a GST tag. GST-PFO stained cholesterol in late endosomes/lysosomes in NPC fibroblasts permeabilized with 0.05% Triton X-100.

GST-PFO can easily be produced in large quantities in *E. coli* and purified by simple one-step column affinity chromatography. The probe is highly selective for cholesterol, as we demonstrated by protein-lipid overlay assay and surface plasmon resonance analysis. The recombinant protein preserves the lytic activity of the native toxin and causes an efflux of 6-carboxyfluorescein from cholesterol-containing liposomes, but not from those devoid of cholesterol (Figure 1B). PFO binds to membranes and causes their permeabilization when the cholesterol content exceeds 30 mol% [25,26]. Therefore, the deposits of cholesterol in NPC cells make them preferable targets of GST-PFO. On the other hand, even though free cholesterol is present in non-NPC cells as well, its level is too low to lead to any marked staining by GST-PFO. The lytic activity of PFO does not affect the staining of cells which were fixed before the treatment with the probe. The presence of the GST tag allows detection of the probe bound to cholesterol in NPC cells with a wide array of anti-GST antibodies. Depending on the label conjugated with the anti-GST antibody various detection techniques can be used. In our hands, the probe allowed unequivocal detection of cholesterol deposits in cells by immunofluorescence and immunoelectron microscopy, and by cellular ELISA. The latter approach is suitable for screening a large number of samples and offers the possibility of a semiquantitative analysis of free cholesterol accumulated in cells. Cellular ELISA based on GST-PFO binding to cells seems to be able to distinguish and quantify “variant” biochemical phenotypes of NPC cells showing the level of cholesterol accumulation in the cells. Our preliminary data show that the GST-PFO probe is also suitable for detection of free cholesterol by FACS analysis (not shown).

The fluorescence technique is routinely used for visualization of free cholesterol deposits in NPC cells by filipin [3,41]. Having a high affinity toward cholesterol, filipin forms fluorescent complexes with the lipid [17]. However, the use of filipin has some drawbacks compared to GST-PFO. The fluorescence of filipin requires UV excitation, is easily scattered, and undergoes rapid photobleaching [18]. The use of GST-PFO for labeling does not pose such constraints, the probe can be detected in a wide range of visible or infrared wavelengths, and the fluorochromes available are fairly stable. Owing to these properties, GST-PFO also facilitates studies on the nature of vesicles harboring cholesterol deposits in NPC cells, which is another advantage of GST-PFO over filipin.

In 2003–2004 Chang’s group made an attempt to use a PFO derivative for cholesterol detection in NPC cells. The so-called BCθ probe was obtained by proteolytic digestion of PFO and biotinylation of the complex of the two resulting fragments [48]. This probe was used to stain a Chinese hamster ovary cell line lacking the NPC1 protein, fibroblasts of NPC patients, and sections of brains of NPC mice. The authors showed that BCθ stained mainly “cholesterol-rich domains” inside the cells [27,28]. The probe entered the cell without prior permeabilization with detergents. Instead, it was shown that the cells became permeable to BCθ when fixed with 4% paraformaldehyde. This approach is unusual, especially taking into account the relatively high molecular mass of BCθ (ca. 57 kDa) and streptavidin-Texas Red (forms tetramers of ca. 76 kDa) used for analysis. In these conditions, both the entry of proteins across the plasma membrane, and particularly across the endosomal/lysosomal membrane and washout of excess protein, are impeded. Perhaps for these reasons the BCθ-positive structures were not well distinguished. In our studies, beside fixation, the cells were additionally permeabilized with 0.05% Triton X-100. Omitting the detergent treatment excluded any labeling of the cells with GST-PFO (ca. 78 kDa). On the other hand, 0.05%-02% Triton X-100 did not solubilize the cholesterol deposits in NPC cells, judging from the intense filipin and GST-PFO staining and cellular ELISA analysis. Permeabilization of cells with Triton X-100 increased the accessibility of cholesterol deposits to GST-PFO. Cholesterol deposits were also freely accessible to methyl-β-cyclodextrin which significantly reduced their level in the cells. This effect was not achieved in non-permeabilized cells exposed to cyclodextrin after fixation with 4% paraformaldehyde [24]. Using permeabilized cells we were able to reveal that vesicles labeled by GST-PFO were also positive for LAMP-1 and LBPA. LAMP-1 is generally accepted as a marker of lysosomes [49] while LBPA accumulates in the inner membranes of multivesicular bodies [43]. In contrast to earlier reports on a high degree of colocalization of filipin-stained vesicles with LBPA [44] in our hands vesicles labeled by GST-PFO displayed significant but not complete overlapping with LBPA staining. This could ensue from technical problems of cell permeabilization which is hard to optimize for both lipids and possibly also from the fact that we work on cells cultured for 72 h in delipidated FBS, which could affect cellular distribution of LBPA. Our data indicate that GST-PFO-positive structures bear markers of late endosomes/lysosomes but not those of endoplasmic reticulum, Golgi apparatus or peroxisomes.

Altogether, the data indicate that GST-PFO is a convenient and reliable probe for detection of cholesterol deposits in cells of NPC patients.

## Abbreviations

BCθ: Biotinylated derivative of perfringolysin O; DOPC: Dioleoylphosphatidylcholine; DPPC: Dipalmitoylphosphatidylcholine; DPPE: Dipalmitoylphosphatidylethanolamine; GST: Glutathione transferase; LAMP-1: Lysosomal-associated membrane protein 1; LBPA: Lysobisphosphatidic acid; LDL: Low density lipoprotein; NPC: Niemann-Pick type C; PDI: Protein disulfide isomerase; PFO: Perfringolysin O; PMP70: Peroxisomal membrane protein of 70 kDa; SM: Sphingomyelin; TEV: Tobacco Etch virus.

## Competing interests

The authors declare that they have no competing interests.

## Authors’ contributions

KK performed confocal and electron microscopy studies, colocalization analysis, participated in drafting and editing the manuscript; EM-S initiated and performed a part of (immuno) fluorescence studies, established conditions for GST-PFO staining of cells; GT and PK cloned GST-PFO and purified the protein, GT also performed cellular ELISA studies; MM and AŁ carried out diagnostics of NPC patients, provided primary cultures of fibroblasts, guided in filipin staining of the cells; MK performed in vitro studies on binding of GST-PFO to cholesterol; AG contributed to studies on LBPA; AS conceived and designed the experiments, analyzed the data, contributed to manuscript writing. All authors read and approved the final manuscript.
